# Surgical treatment of permanent atrial fibrillation during cardiac surgery using monopolar and bipolar radiofrequency ablation

**Published:** 2003-07-01

**Authors:** Stephan Geidel, Jorg Ostermeyer, Michael Lass, Sigrid Boczor, Karl-Heinz Kuck

**Affiliations:** * Department of Cardiac Surgery, AK St.Georg, Lohmuhlenstraße 5, 20099 Hamburg, Germany; † Department of Cardiology, AK St.Georg, Lohmuhlenstraße 5, 20099 Hamburg, Germany

**Keywords:** Atrial fibrillation, atrial fibrillation surgery, radiofrequency ablation, arrhythmia surgery, heart valve surgery, maze surgery, bipolar radiofrequency ablation

## Abstract

**Objective:**

Permanent atrial fibrillation (pAF) is a serious problem in cardiac surgery: An incidence of 3.5% among all patients scheduled for open heart surgery, 9.8% in heart valve cases and 45.6% among patients with severe rheumatic mitral valve (MV) disease was observed in our institution. Our experience with radiofrequency (RF) ablation procedures to treat pAF in these cases is reported.

**Methods:**

Since February 2001 monopolar endocardial RF ablation procedures creating two encircling isolation lesions around the left and the right pulmonary veins (LPVs, RPVs) and a connection line between both were performed in patients with pAF concomitant to heart valve surgery. Since March 2003 bipolar RF ablation was used as an adjunct to CABG surgery. Amiodarone was given for 3 months after surgery.

**Results:**

Sixtytwo patients with pAF underwent surgical ablation procedures and primary valve (mitral: n=45; aortic: n=13; aortic+mitral: n=1; LA-diameter 55.6±7.3 mm) or CABG surgery (n=3). Follow-up was performed at 3, 6, 9, 12, 18 and 24 months; 75% had stable sinus rhythm (SR) at late follow-up. Almost 90% of the patients with a preoperative LA-diameter of <56mm had SR.

**Conclusion:**

Isolation of the PVs using RF ablation procedures in combination with amiodarone therapy represents a safe and efficient option to cure pAF in patients undergoing open heart surgery.

## Introduction

In cardiac surgery strategies to treat permanent atrial fibrillation (pAF) effectively are of greatest interest because pAF is a frequent problem that substantially deteriorates the prognosis [1-3]. The data concerning the incidence of pAF following the classification of the ACC/AHA/ESC Practice Guidelines [4] that had been documented to persist for a period of at least 6 months among all patients scheduled for open heart surgery in our institution between February 2001 and April 2003 are outlined in Table 1.

Since Cox has demonstrated that AF can be definitely eradicated [5], efforts were made to achieve alternative and particularly less complex methods using surgical catheter ablation techniques during cardiac surgery. A broad spectrum of these methods has been presented recently [6-14]. Our experience with radiofrequency (RF) ablation procedures to treat pAF during open heart surgery is reported.

## Material and methods

Etiology of heart valve disease was assessed by clinical history, intraoperative valve examination and histological analysis. Patients hospitalized for heart valve surgery with pAF (≥6months) underwent combined intraoperative AF treatment with monopolar RF ablation. Since March 2003 a bipolar RF ablation procedure was performed in CABG cases with pAF. Patients with any other form of AF (intermittent or pAF <6months), emergency operation, severe reduced left ventricular function (EF≤25%), acute bacterial endocarditis, cachexia [Body Mass Index (BMI) ≤24], severe intracavitary thrombosis or extreme left atrial size were excluded (LA diameters of ≥72mm were assessed to be our limit for the procedure).

### Surgical procedure

#### Heart valve cases

**Figure 1:**
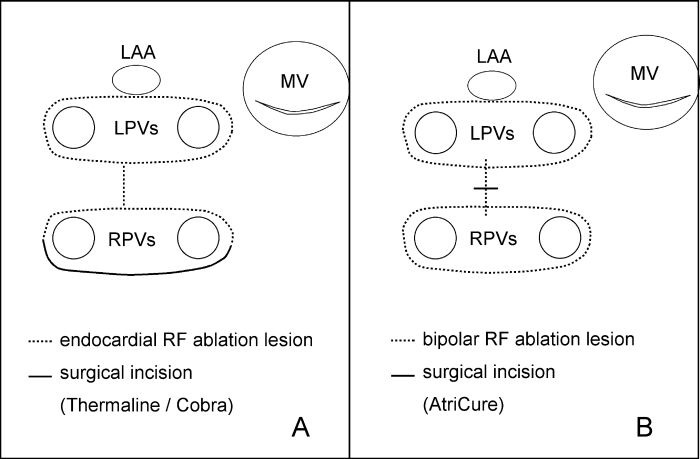
Ablation schemes. LAA, left atrial appendage; LPVs, left pulmonary veins; RPVs, right pulmonary veins; MV, mitral valve.

The surgical procedure has been described detailed elsewhere [15]. To create endocardial RF ablation lesions two almost identical systems were used: either the Thermaline® device or (since 01/02) the Cobra® device (both Boston Scientific Corporation, San Jose, USA). Monopolar RF ablation was performed using 100W RF power for 120 sec, the local temperature was set at 70°C. The first lesion line completed the isolation of the right pulmonary veins (RPVs) from the inferior to the superior RPV using the left atriotomy. Isolation of the left pulmonary veins (LPVs) was performed with a semicircular ablation line close to the inferior, and another one around the superior LPV. These were connected by a transverse lesion across the posterior wall of the LA (Figure 1A). Arrangements to avoid thermic esophageal injury were: (1) cachectic patients were excluded, (2) a dry compress was passed behind the LA before delivery of RF energy, (3) the transesophageal echocardiogram (TEE) probe was removed during the ablation procedure, (4) a flexible ablation probe was used and adapted to the tissue without pressure, (5) local temperature was set at 70°C and (6) monopolar RF ablation was performed precisely under direct view during conventional open heart valve surgery only.

#### CABG cases

The bipolar AtriCure device (AtriCure Inc., Cincinnati, USA) was used for an almost identical lesion pattern (Figure 1B). The device consists of a hand piece, a footswitch, connecting cables and an ablation and sensing unit (ASU) that (1) delivers RF energy while simultaneously measuring the tissue conductance and (2) uses a temperature sensing mechanism (range of 45 - 55°C). During ablation the tissue is impacted between two jaws of the hand piece and energy is delivered by footswitch. The ablation was finished when the ASU monitor indicated that the tissue conductance was at least 3 sec below 2.5 Millisiemens. After start of cardiopulmonary bypass isolation of the RPVs and the LPVs was performed by grasping the adjacent atrial tissue. Then a purse-string suture with a tourniquet was set at the posterior wall of the LA. The distal jaw was inserted through a small incision in direction of the LPVs and RF ablation was performed after clamp-closure, then the distal jaw was inserted in direction of the RPVs and the connection line was completed (Figure 1B).

### Perioperative management, follow-up and statistical analysis

The perioperative management has been described detailed elsewhere [15]. Standard 12-lead electrocardiogram (ECG) and transthoracic echocardiogram (TTE) were routinely performed on admission (evaluation of LA diameter) and before discharge. Administration of amiodarone was started before end of cardiopulmonary bypass and given for 3 months after surgery. Early recurrence of AF was DC cardioverted after saturation with amiodarone. Patients with CABG, mitral valve (MV) repair or bioprosthesis got cumarine for 3 months, patients with mechanical valves lifelong anticoagulation. All patients were restudied 3, 6, 9, 12, 18 and 24 months after surgery by standard 12-lead ECG and clinical examination. Quantitative preoperative and operative data were normally distributed and described by arithmetic mean ± standard deviation; qualitative distributed data were presented as absolute frequencies. For pAF and sinus rhythm (SR) the relative frequency among all patients and some subgroups were calculated. Qualitative characteristics were compared using the exact Fisher Chi-Square-Test. All p-values were two-tailed and interpreted nominal that is not adjusted for multiple comparisons. P-values <0.05 were considered to be statistically significant. Analysis was performed with SPSS for Windows 11.5.1.

## Results

### Heart valve cases

Fifty-nine patients underwent surgical pAF ablation procedures associated with primary valve operations (Table 2). From these patients 45 had MV, 13 AV and 1 AV+MV surgery; 13 were excluded. The main group suffered from rheumatic heart valve disease (39/59). Mean LA diameter was 55.6±7.1mm, 32 patients had a small (<56mm) and 27 patients a large LA (≥56mm).There were two cases of hospital mortality (3.4%; 1 cardiac, 1 non-cardiac). Mean follow-up time up to April 2003 was 14.1 ± 9.9 months (Table 3). One-year-survival was 91.7%; late mortality was related to cancer disease (n=2) and sudden death (n=1). At late follow-up approximately 75% of all patients and almost 90% of those with a preoperative LA-diameter of <56mm were in stable SR. Early postoperative recurrence of AF during the first 3 months (32/59) was DC cardioverted in 13 of 25 cases, 6 turned to SR spontaneously later. Preoperative duration of AF was not predictive for long term results.

### CABG cases

In 3 bipolar RF ablation cases (Table 2) no severe complication occurred. At end of surgery 2 patients were externally paced in DDD-mode, one was in SR. Early postoperative return of AF was DC cardioverted in 1 of 2 cases; 2 of 3 patients left the hospital in SR.

## Discussion

### Rationale of surgical practices to treat pAF

Reliable and effective treatment strategies for pAF are of greatest interest in cardiac surgery because pAF causes two times higher rates of death, five times higher risk of stroke, reduced cardiac output and the need of systemic anticoagulation with the danger of bleeding [1-3]. The incidence among patients scheduled for open heart surgery is particularly high among heart valve cases and of special importance in MV disease [15]. That is why most of the recently reported surgical AF ablation data were observed following heart valve (particularly MV) surgery [6-14]. The permanent form of AF is one of two AF-types (permanent and intermittent) and normally the final mode in which non-permanent AF cumulates [4]: patients with pAF have atrial fibrillation all of the time without any episode of SR. The variant clinical presentations of permanent and intermittent AF have unfortunately caused heterogeneous nomenclatures in different countries with currently used misleading terms (chronic, paroxysmal or persistent AF) and the resulting difficulty to compare the success-rates of different groups.

During the past years mainly three different energy sources have been favoured for surgical practice to create atrial lesions to cure these AF-types; RF energy has been established for variant treatment strategies and became the most widely used energy source for AF surgery [8-12]. The use of microwave [13,14] and cryoablation [6] has been described alternatively. In these investigations [8-12] RF energy was used without exception in monopolar fashion for endocardial or/and epicardial ablation techniques with more or less comparable and successful results. However, two theoretical deficiencies remain with the application of monopolar RF energy: (1) transmurality of the created lesions is not definitely guaranteed and (2) rare but fatal complications because of too deep lesions have occurred [16,17]. Experiences with possibly reliable bipolar RF ablation techniques that guarantee lesion transmurality and continuity and definitely bar extra-cardiac tissue injury are therefore of great interest.

Many surgeons who used ablation techniques in the past followed more or less closely the principles of the Maze procedure which are PV isolation, reduction of atrial size and block of re-entrant circuits by complex incisions [8,9,11,12]. An ideal lesion pattern should combine (1) slight invasiveness, (2) simplicity, (3) high reproducibility and (4) saving of time with excellent success rates in almost all cases. The question if such a lesion pattern is actually applied in practice must be answered negative. The mechanisms of AF initiation and maintenance obviously vary and are connected with individual electrophysiological and pathological atrial tissue changes in cases with AF and particularly pAF [18-20]. It is possible that individual patients with pAF probably need individual surgical ablation procedures. The relevant questions are: (1) does a lesion pattern exist which can be recommended as a basis and can be completed easily according to the individual pathological or electrophysiological substrate and (2) can patients be defined who need specific additional ablation procedures?

Referring to this Haissaguerre has described an important pathophysiologic finding: he demonstrated that the initiation of AF originates from rapidly firing foci predominantly located inside the PVs [21]. According to that the concept was developed that isolation of the PVs creating transmural encircling RF ablation lesions around the LPVs and the RPVs should be a sufficient basis for surgical ablation procedures. To save LA function and to bar potential generation of foci the maze pattern of multiple incisions was reduced to a short connection line between both. During valve surgery RF ablation was performed from the endocardial side with direct view on the atrial tissue to guarantee continuity of the lesions. A new bipolar RF approach was recently used in primary CABG cases with pAF as an endocardial proceeding would have been of obvious disadvantage because of greater invasiveness and prolonged aortic cross clamping time in these cases. Amiodarone was normally given to reduce postoperative recurrence of AF [22].

### Evaluation of the results

Our results conform to basic experiences of other research [5,21,23,24]: AF wavelets sustained by foci located inside the PVs were blocked by the created lesions, the described antiarrhythmic protection supported SR [22] during the unstable initial stage, which was approximately 3 months. Early recurrence of AF after ablation surgery obviously occurred due to the fact that the refractory period of the atrium was still shortened. In the case of AF recurrence DC cardioversion was still recommended, so the influence on the long-term results must be further clarified. Our data indicate that the preoperative LA size is of significant concern for the success of the described method. However, it remains uncertain whether the LA size itself is the critical issue. It can be expected that rather cellular, structural morphologic and in parallel electrophysiological changes of the atrial tissues are more marked in cases with progressive enlargement and hypertrophy of the atria [18,19,20]; this electrical and anatomic atrial remodeling is supposed to be the reason for what has been decscribed as AF begats AF [25]. It must be conceded that an additional effect may be that in cases with small LA the encircling lesions possibly encompassed larger portions of the LA in comparison to its overall size than in patients with large LA; in that cases re-entrant circuits in the LA tissue could be interrupted more frequently. Besides LA reduction has been described to be of advantage for restoring SR in patients with chronic AF and large LA following MV surgery [26].

Even if our patients were approximately a decade older compared to patients of other studies [9,10] surgery was tolerated well, particularly no case of esophageal injury [16,17] was observed. Also confirmed was the increase of SR during the following months after surgery [8-11]. It can be suggested that a 3 months administration of amiodarone is appropriate but should be handled flexible. The described procedures are of slight invasiveness and easy to perform. The lesion pattern can be created with monopolar RF energy and with a bipolar approach as well. However, it will have to be shown, if the results with bipolar RF energy are as encouraging as the described data in valve patients. As for MV surgery the LA has to be opened anyhow we continue to recommend for the present an endocardial ablation technique for these patients.

### Limitations

For rhythm evaluation only 12-lead ECG was used. We consider to complete the follow-up data by performing a 24-hour-ECG registry to assess the possibility of nonpermanent AF. The data were not evaluated under randomized conditions.

## Conclusion

We suppose that the described concept provides a successful treatment of pAF in patients undergoing open heart surgery; it fulfils all demands of an effective and easy to handle method. It simplifies the treatment of pAF and can be recommended in patients undergoing cardiac surgery. The advantages compared to other techniques are: (1) the procedure is easy to practice and (2) atrial tissue trauma is extremely slight. Our data indicate that particularly in patients with small LA restoration of stable SR in 90% of the cases can be achieved.

## Figures and Tables

**Table 1:**
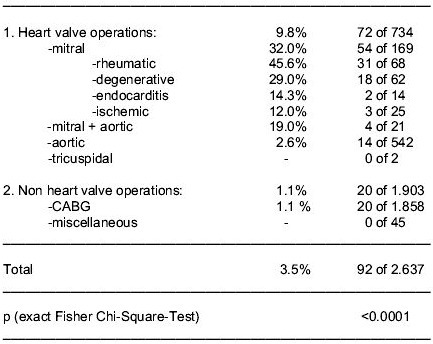
Incidence of pAF (6 month) among 2,637 open heart cases

**Table 2:**
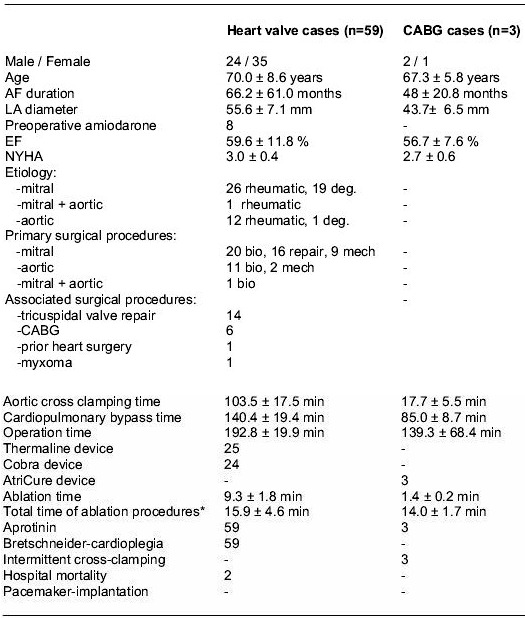
Preoperative and operative data (n=62)

*: including adjustment of the equipment and a precise adaption of the probe to the tissue (Thermaline / Cobra) and preparation of the PVs (Atricure); deg, degenerative MV disease; bio, bioprosthesis; mech, mechanical valve.

**Table 3:**
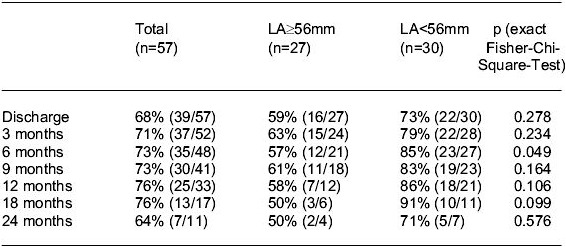
Follow-up data: Cases with stable SR after heart valve surgery and RF ablation procedures
